# Successful pancreatoduodenectomy of *de novo* duodenal malignancy after orthotopic liver transplantation: A case report

**DOI:** 10.3389/fsurg.2022.1068215

**Published:** 2023-01-06

**Authors:** Linhua Ji, Danhua Xu, Chunchao Zhu, Jia Xu, Hui Cao, Gang Zhao

**Affiliations:** Department of Gastrointestinal Surgery, Ren Ji Hospital, School of Medicine, Shanghai Jiao Tong University, Shanghai, China

**Keywords:** *de novo* malignancy, duodenal carcinoma, liver transplantation, pancreatoduodenectomy, case report

## Abstract

**Introduction:**

Liver transplantation is a risk factor for premalignant and malignant changes of the duodenum. De novo duodenal malignancy is seldom reported after liver transplantation.

**Case Report:**

The present study reports a case of an asymptomatic 67-year-old male patient who underwent liver transplantation more than 10 years ago and subsequently developed duodenal malignancy. Endoscopic biopsy of the *de novo* duodenal malignancy indicated duodenal carcinoma and pancreatoduodenectomy (PD) was performed. The patient was successfully discharged 12 days after the surgery. A metastatic lesion occurred at the right seventh rib 14 months after the pancreatoduodenectomy. Postoperative pathological examination indicated hepatocellular carcinoma metastasis.

**Conclusions:**

To the best of our knowledge, this case type has not been previously reported. The present study sheds light on the development, the treatment, the prognosis, and the management of a new type of *de novo* duodenal malignancy.

## Introduction

De novo gastrointestinal malignancies are common after organ transplantation, which are often diagnosed with an advanced stage and have poor prognosis. However, *de novo* duodenal malignancy after liver transplantation has not been previously reported.

We report the case of an asymptomatic male patient with a history of liver transplantation who subsequently developed *de novo* duodenal carcinoma. The patient then underwent surgical resection and was given postoperative chemotherapy. The study involving human participant was reviewed and approved by the Research Ethics Committee of Ren Ji Hospital, School of Medicine, Shanghai Jiao Tong University and was carried out in accordance with the ethical standards of the World Medical Association's Declaration of Helsinki. The patient provided written informed consent to participate in this study. The purpose of this study was to share the experience on how to monitor and manage this new type of *de novo* duodenum malignancies in patients who have received liver transplantation. The complex nature of such malignancies requires that a systematic, multidisciplinary approach be undertaken.

## Case presentation

A 63-year-old male patient was admitted to the Department of Gastrointestinal Surgery, Ren Ji Hospital, Shanghai Jiao Tong University (Shanghai, China) on May 22, 2018. The patient had undergone appendicectomy 30 years ago and orthotopic liver transplantation on January 28, 2011 due to hepatocellular carcinoma with decompensated liver cirrhosis of hepatitis B. During the operation, we dissected the first hepatic hilar region and cut off the common hepatic duct above the junction of cystic duct. We cut off the left and right hepatic arteries above the bifurcation respectively. We dissected and cut off the portal vein, the infrahepatic and suprahepatic vena cava after blocking the blood flow. Then we reconstructed the inferior vena cava, portal vein, artery and biliary duct between donor liver and recipient liver. Postoperative pathology revealed hepatocellular carcinoma in the native liver. Upon further questioning, the patient admitted weight loss about 7.5 kg (from 72.5 kg to 65 kg) during the last several months but denied any history of visible gastrointestinal bleeding. The patient had no noticeable family history, nor did he have a history of smoking, alcohol drinking, or toxic environment exposure.

The patient had been taking tacrolimus (3 mg), CellCept (0.5 g), entecavir (0.5 mg) after liver transplantation every day, and his blood concentration of FK506 was maintained at about 2.3 ng/ml. Routine blood and liver function tests were both normal, and the results of AFP, CEA, and hepatitis B virus tests were all negative. However, the CA19-9 level was moderately elevated (68.24 U/ml, compared to a normal range of up to 33 U/ml).

**Figure 1:**
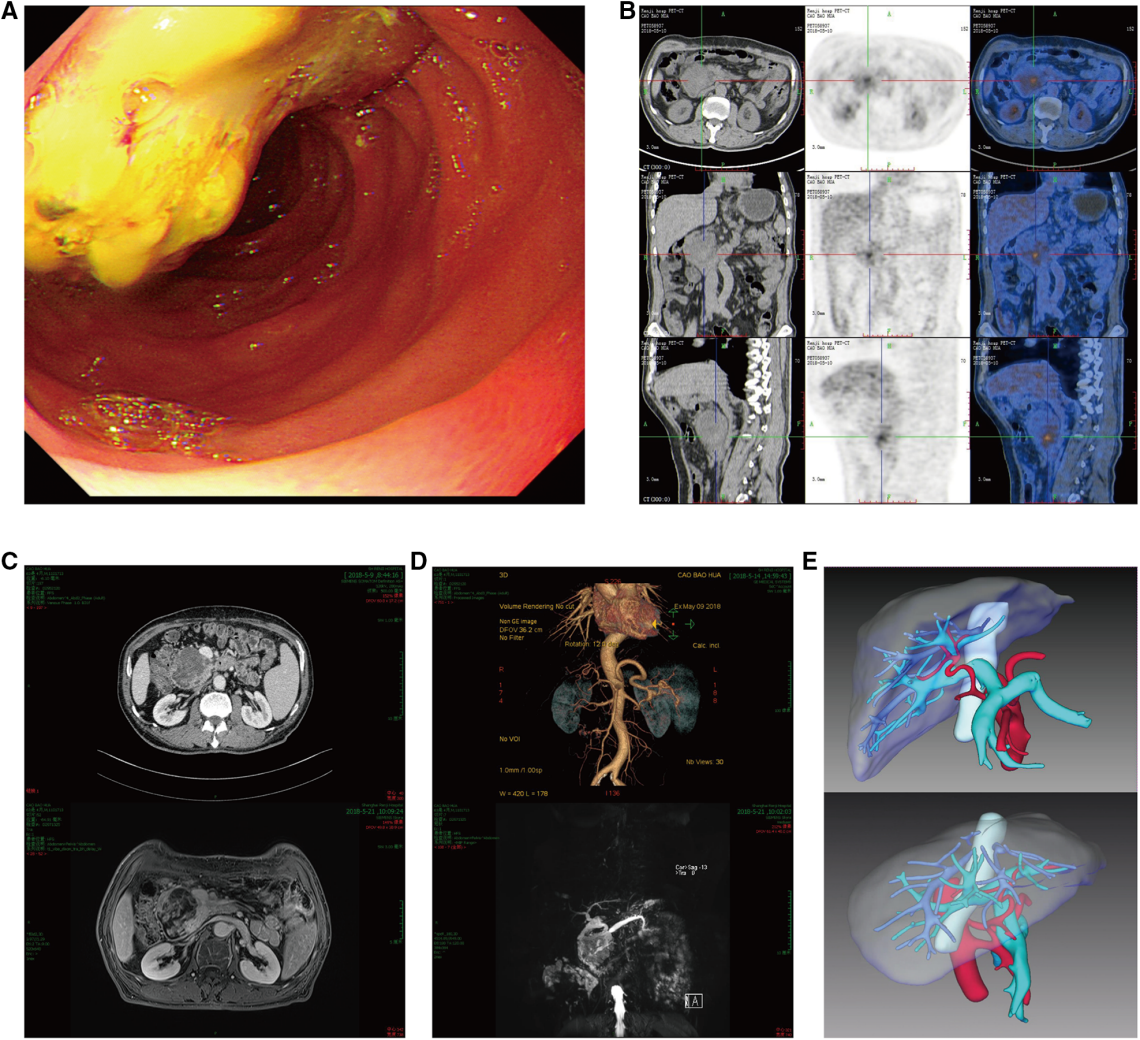
(**A**) gastroduodenoscopy revealed a mass lesion located in the descending part of the duodenum. (**B**) PET-CT scan showed a tumor in the duodenum with SUVmax = 5.5, its size was about 6.4 × 6.7 cm. (**C**) Contrast-enhanced computed tomography (CT) and magnetic resonance imaging (MRI) detected a tumor of approximately 6.5 cm in diameter that was located in duodenum. (**D**) Magnetic resonance cholangiopancreatography (MRCP) found that duodenal tumor caused dilation of extrahepatic bile duct and pancreatic duct. (**E**) CT angiography and three-dimensional reconstruction revealed donor liver's artery went from the back down side of portal vein to the front right side of it, the gastroduodenal artery is visible.

Gastroduodenoscopy revealed a mass lesion located in the descending part of the duodenum (Figure 1A). Positron emission tomography-computed tomography (PET-CT) scan showed a mixed density mass in the descending part of the duodenum, and its metabolism of fluorodeoxyglucose (FDG) increased unevenly (SUVmax = 5.5) (Figure 1B), and no metastasis to other organs. Contrast-enhanced computed tomography (CT) and magnetic resonance imaging (MRI) detected a tumor of approximately 6.5 cm in diameter that was located in duodenum (Figure 1C). Further, Magnetic resonance cholangiopancreatography (MRCP) found that the duodenal tumor caused dilation of extrahepatic bile duct and pancreatic duct (Figure 1D). Tissue biopsy was then obtained and pathologically examined. The results indicated mixed adenocarcinoma (tubular adenocarcinoma and mucinous adenocarcinoma). In addition, CT angiography with three-dimensional reconstruction was performed to reveal the anatomical structure. From the reconstructed liver structure, it can be seen that the donor liver's artery went from inferior and posterior side of portal vein to superior and anterior side of it. Also, the gastroduodenal artery was clearly visible (Figure 1E).

**Figure 2:**
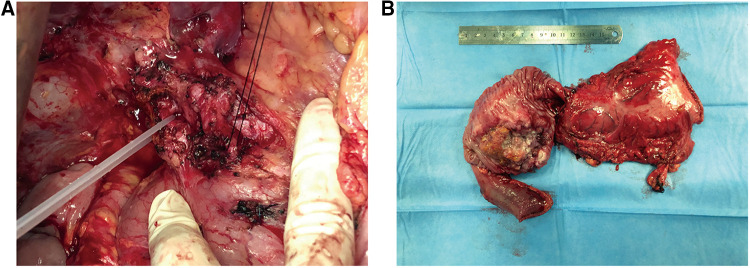
(**A**) intraoperative anatomy of the donor liver's artery and gastroduodenal artery. (**B**) By gross appearance, the resected specimen shows a tumor with ulceration measuring 6.5 × 5 × 5 cm.

Because the patient was in good physical condition and pre-operative imaging suggested the disease as resectable, pancreatoduodenectomy (PD) was performed and complemented with pancreatojejunostomy Roux-en-Y anastomosis with peripancreatic lymph nodes dissection and vascular dissection (Figure 2A). Intraoperative examination revealed no peritoneal dissemination. The operation lasted for nearly 10 h and intraoperative blood loss was about 500 ml. The patient was transferred to the intensive care unit (ICU) after surgery and was discharged in 12 days. 4 weeks after the operation, the patient was given 4 cycles of oral chemotherapy (days 1–21,S-1/ Tegafur, Gimeracil and Oteracil Potassium Capsules, 50 mg orally B.I.D; cycle repeated every 28 days). Following the pancreatoduodenectomy, the patient continued tacrolimus usage daily and his blood concentration of FK506 was maintained at about 3 ng/ml.

The gross appearance of the resected specimen was a tumor with ulceration measuring 6.5 × 5 × 5 cm (Figure 2B). Postoperative pathological examination showed mixed duodenal adenocarcinoma (tubular adenocarcinoma and mucinous adenocarcinoma) with medium differentiation. The tumor had infiltrated the full wall of the duodenum and invaded the pancreas and part of the bile duct. Resection margins and lymph nodes dissected were both negative. According to the American Joint Committee on Cancer (AJCC) Cancer Staging Manual (Springer International Publishing, 8th Edition 2018), the tumor was classified as stage IIB (T4N0M0).

**Figure 3:**
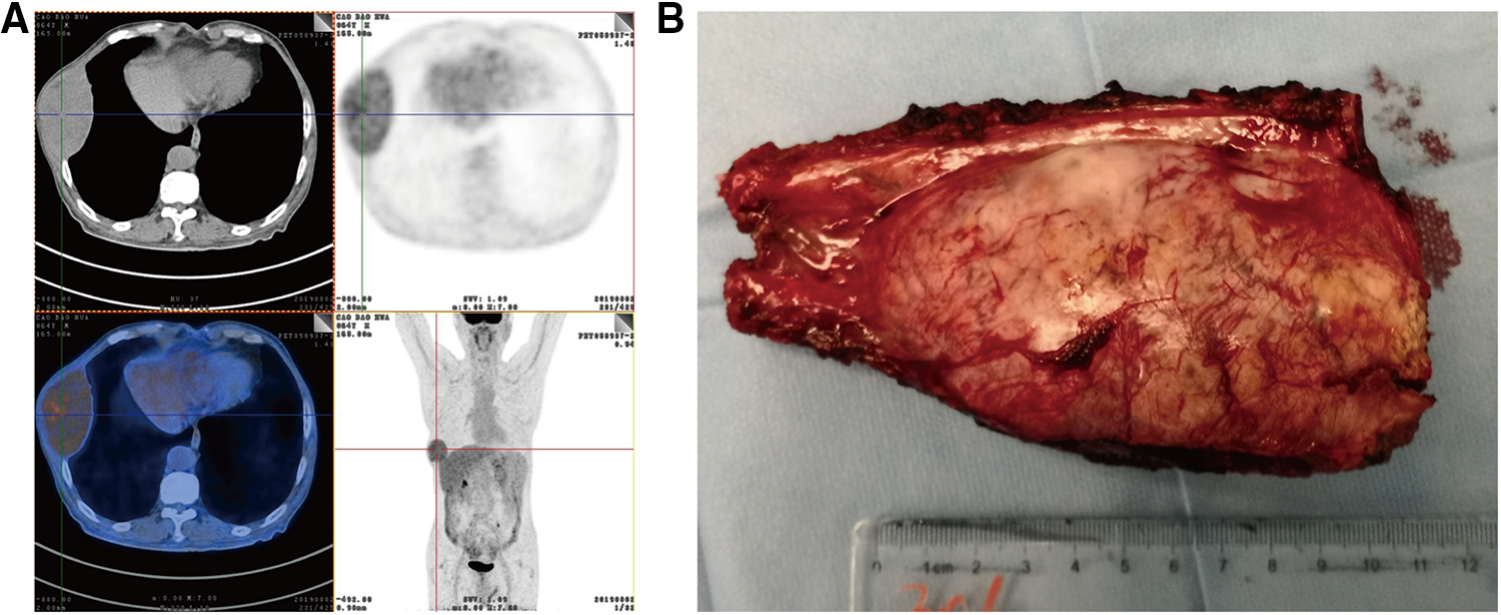
(**A**) PET-CT detected a tumor metastasis of approximately 8.9 cm × 5.3 cm that was located in right seventh rib. (**B**) By gross appearance, the resected specimen shows a metastatic tumor measuring 11 cm × 7 cm × 5 cm.

**Figure 4:**
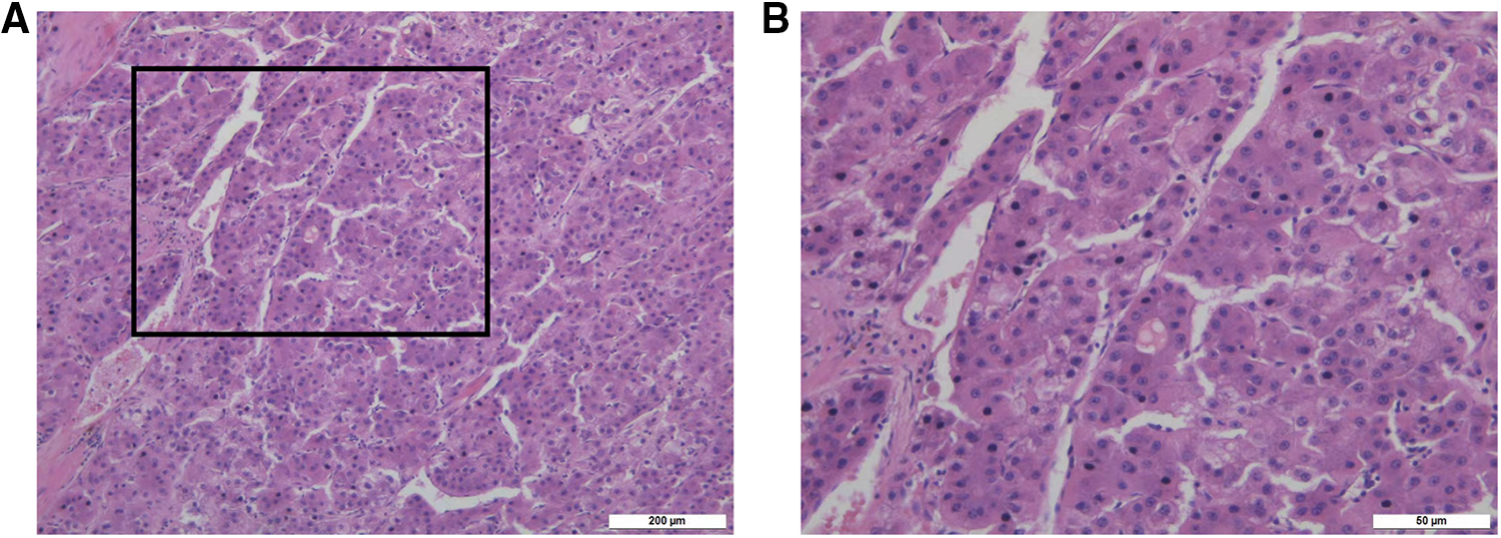
(**A**) the pathological picture of tumor in right seventh rib confirmed hepatocellular carcinoma metastasis under 10× microscope. (**B**) The pathological picture of tumor in right seventh rib confirmed hepatocellular carcinoma metastasis under 40× microscope.

After 13 months of regular follow-up, there was no clinical or radiological evidence of recurrence. CA19-9 was dropped and remained low. In July 2019, however, MRI and PET-CT scan indicated a metastatic lesion of approximately 8.9 cm × 5.3 cm located at the right seventh rib (Figure 3A). At that time, AFP, CEA and CA19-9 were not elevated. We performed surgical resection of the metastatic lesion in the rib. First we confirmed that the right lung was not invaded by the tumor. Then we cut off the right 7th and 8th ribs, completely remove the tumor, and sutured the intercostal artery. We reconstructed the bone structure of chest wall with matrix steel plates. The gross appearance of the resected specimen was a tumor measuring 11 cm × 7 cm × 5 cm (Figure 3B). Postoperative pathological examination of the tumor specimen indicated hepatocellular carcinoma metastasis (Figures 4A,B). By August 2022, CT didn't show evidence of recurrence. AFP, CEA and CA19-9 were maintained within normal limits.

## Discussion

Organ transplantation is an effective method to treat patients with organ failure or end-stage organ disease. By recovering normal physiological function, organ transplantation can help improve life quality of the patients. At present, liver and kidney transplantations are the most widely adopted. Cornea, pancreas, small intestine, uterus and combined heart-lung transplantation are also conducted in clinical practice.

Immunosuppressive agents are routinely administered after organ transplantation, which help improve the long-term survival rate. As an undesired consequence, the risk of *de novo* malignancies increases as a patient's survival increases. The concept of *de novo* malignancy was first proposed by Starzl in 1968 and refers to a malignancy developed after organ transplantation which is not caused by the primary disease. De novo malignancy has now ranked the second most common cause of death after organ transplantation. It is estimated that, over the next 20 years, *de novo* malignancies including gastrointestinal malignancies will become the primary cause of death for transplant recipients, surpassing cardio-cerebrovascular complications (1).

De novo gastrointestinal malignancies are common after liver or kidney transplantation and are usually at advanced stages when diagnosed. As such, these malignancies have poor prognosis. According to the latest statistics from the National Cancer Registry Center in 2016, the incidences of gastric and colorectal cancers in China in 2012 were 31.28/100,000 and 24.47/100,000, respectively. The mortality rates of these two types of cancer were 22.04/100,000 and 11.77/100,000, respectively (2). However, reports from several research centers showed that the incidences of *de novo* gastric and colorectal malignancies after liver transplantation were 110/100,000–240/100,000 and 170/100,000–860/100,000, respectively (3). Apparently, the incidence of *de novo* gastrointestinal malignancies in transplant recipients was much higher than that in the general population. Further, studies have demonstrated that the onset age of lung, breast, prostate and colon malignancies in liver transplant recipients were earlier than that in the general population (4). In addition, the survival rate of *de novo* gastric malignancy in liver transplant recipients was lower than that in the general population. A retrospective study involving 2,000 liver transplantation patients showed that the 5-year survival rate for gastric cancer was significantly lower in the study cohort vs. the general population (0% vs. 30%) (5). De novo gastrointestinal malignancies can be challenging in surgical treatment, and early diagnosis of such malignancies after organ transplantation can help improve the prognosis (6).

Factors that may contribute to the development of *de novo* malignancies in transplant recipients include age, direct or indirect effects of immunosuppressive agents, precancerosis, and survival time of transplanted grafts and recipients, etc. For example, transplant recipients over 40 years old is one significant risk factor of *de novo* malignancies (7). Long-term postoperative use of immunosuppressive agents may facilitate tumorigenesis by suppressing the transplant recipient's immune system and allowing tumor cells to escape immune surveillance and proliferate. Additionally, a suppressed immune system is often associated with other undesired consequences such as infection and insulin resistance, which may promote the proliferation of tumor cells.

Furthermore, some immunosuppressive agents such as calcineurin inhibitor (CNI) are carcinogenic and should be used with caution, for example, as a combination therapy with an anti-tumor agent like mycophenolate mofetil (MMF) or mammalian rapamycin target protein inhibitors (mTOR inhibitors) (8–10).

Gastric tumorigenesis is often correlated with Helicobacter pylori infection. Chronic gastritis, gastric ulcer and gastric polyp are precancerous lesions of gastric cancer. Presence of such risk factors also increases the incidence of *de novo* gastric cancer after organ transplantation. Similarly, primary sclerosing cholangitis, ulcerative colitis and colonic polyps are risk factors of colorectal cancer and may increase the incidence of colorectal cancer after organ transplantation (11–14).

De novo gastrointestinal malignancies have no specific clinical manifestations compared to common gastrointestinal malignancies. As such, transplant recipients who are at elevated risks of cancer should be carefully monitored and avoid exposure to carcinogens. Any precancerous conditions identified should be examined and treated promptly. In addition, the use of immunosuppressive agents should be carefully tailored such that a minimal dosage is applied while the graft function is effectively maintained. For example, in patients with stable postoperative organ function and without acute or chronic rejection, the dosage of immunosuppressive agents may be reduced gradually. Liver transplant recipients can greatly benefit from immunosuppression weaning (15).

In this particular case, previous orthotopic liver transplantation didn't alter the anatomy of hepatic pedicle, such as the hepatic artery, the common bile duct, or the portal vein. However, liver surgery and long-term postoperative use of immunosuppressive agents led to severe adhesion in the patient's hepatic hilar region, which significantly increased the difficulty of surgical operation on the *de novo* duodenal tumor. Meticulous care must be taken during the dissection of the hepatic hilar region and the resection of lymph nodes in order to avoid injuries to the hepatic artery, the common bile duct, or the portal vein, which might result in postoperative hepatic dysfunction or even hepatic failure. Postoperative management of the patient was also challenging because the use of adjuvant chemotherapy agents must be considered in conjunction with that of immunosuppressive agents. To balance the need for both immunosuppression and adequate immunity, the chemotherapy regimen including the selection of agents and the number of cycles must be carefully evaluated.

In our study, we reported for the first time a patient who developed *de novo* duodenal cancer 7 years after liver transplantation. After surgical resection of the *de novo* duodenal tumor and adjuvant chemotherapy, the patient remained disease free for 13 months, at which point recurrence developed in the form of a metastatic lesion at the rib. Pathological examination of the metastatic tumor indicated hepatocellular carcinoma metastasis, which was probably caused by implantation metastasis or hematogenous metastasis.

In conclusion, the morbidity and mortality rates of *de novo* gastrointestinal malignancies after organ transplantation are higher than primary malignancies. Specifically, liver transplant recipients may develop cancer at various locations including uncommon ones like reported in the present study. Therefore, these patients should be carefully surveyed in order to facilitate early diagnosis and improve prognosis.

## Data Availability

The original contributions presented in the study are included in the article/Supplementary Material, further inquiries can be directed to the corresponding author/s.
